# Taxonomic revision of *Ectyphus* Gerstaecker, 1868  and *Parectyphus* Hesse, 1972 with a key to world Ectyphinae (Insecta, Diptera, Mydidae)

**DOI:** 10.3897/zookeys.73.840

**Published:** 2010-12-29

**Authors:** Kathleen M. Lyons, Torsten Dikow

**Affiliations:** 1 3307 West 97th Street, Evergreen Park, IL 60805, USA; 2 Biodiversity Synthesis Center, Field Museum of Natural History, 1400 South Lake Shore Drive, Chicago, IL 60605, USA

**Keywords:** Mydidae, Ectyphinae, Afrotropical, *Ectyphus*, *Parectyphus*, world key

## Abstract

The Afrotropical Mydidae genera Ectyphus Gerstaecker, 1868 and Parectyphus Hesse, 1972 are revised. Six species of Ectyphus are recognised (Ectyphus abdominalis Bezzi, 1924, Ectyphus armipes Bezzi, 1924, Ectyphus capillatus Hesse, 1969, Ectyphus pinguis Gerstaecker, 1868, and Ectyphus pretoriensis Bezzi, 1924), of which one is newly described from Kenya, Ectyphus amboseli **sp. n.** Two species, Ectyphus bitaeniatus Hesse, 1969 and Ectyphus flavidorsalis Hesse, 1969, are newly synonymised with Ectyphus pinguis. The monotypic genus Parectyphus Hesse, 1972 and the male of its type species Parectyphus namibiensis Hesse, 1972 are re-described while the female is described for the first time. Comments on the distribution of all species within biodiversity hotspots are given. A dichotomous identification key to the genera and species of world Ectyphinae is provided and illustrated keys to the world Ectyphinae are made available online in both dichotomous and multi-access, matrix-based formats.

## Introduction

**Figure 1.**
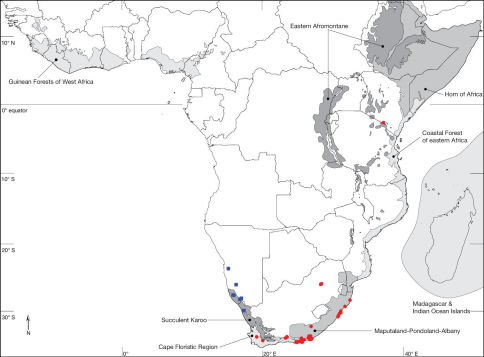
Map of the Afrotropical Region with biodiversity hotspots *sensu* Conservation International showing distribution of Ectyphus (red circles) and Parectyphus (blue squares). Note the distribution of Ectyphus in south-eastern Kenya.

Mydidae is one of the smaller families of Diptera, with 471 species currently described in 66 genera world-wide. Mydids are infrequently collected, so little is known about the life history and seasonality of these interesting flies. Current knowledge of the Mydidae fauna indicates that most of the species diversity occurs in the Afrotropical Region, specifically in Namibia and western South Africa. To this day, the subfamily Ectyphinae is represented by two groups of geographically isolated genera: Heteromydas Hardy, 1944 and Opomydas Curran, 1934 from western North America (Mexico: Baja California Norte, Baja California Sur, Sonora and the USA: Arizona, California, Nevada, New Mexico, Texas), and Ectyphus Gerstaecker, 1868 and Parectyphus Hesse, 1972 from southern Africa (Namibia and South Africa). The objective of this study is the revision of the two Afrotropical genera, including the description of the first species to be collected in eastern Africa (Fig. 1). The revision of Ectyphus and Parectyphus is based on 131 and 11 specimens, respectively, entails the presentation of identification keys and descriptions of all species of the two genera, and summarises what is known about their biology and distribution. In addition, keys for the identification of all known genera and species are provided. For regularly updated distribution maps for all Mydidae species based on specimen occurrence data see http://www.mydidae.tdvia.de/mydidae_specimen_map.

## Taxonomic history of Ectyphus and Parectyphus

Gerstaecker (1868) erected the genus Ectyphus and described the type species Ectyphus pinguis from ‚Africa australis‘. His diagnosis of the genus was based on the presence of a small ‚posterior crossvein‘ (= M_3_+CuA_1_) on the wing, a circlet of spurs on the female ovipositor (acanthophorite spurs), and a reduced, rudimentary proboscis. These characters are not autapomorphies for Ectyphus, but are also found in genera of other subfamily taxa.

(Williston (1886, 1898) described Ectyphus limbatus Williston, 1886 from Arizona and Ectyphus townsendi Williston, 1898 from New Mexico, USA, and Séguy (1928) described Ectyphus athamas from ‘Basse California’ (= Lower California) in present-day Baja California Norte state, Mexico. Bezzi (1924), when studying the southern African Mydidae, suggested that the North American Ectyphus belong to a separate genus based on the lack of a metathoracic tibial spine in females. Curran (1934) then erected the genus Opomydas to include these North American species, leaving only African species in Ectyphus. Kondratieff and Fitzgerald (1996) provide a recent revision of the North American species.

Bezzi (1924) described two new South African species of Ectyphus, *i.e.*, Ectyphus abdominalis from Montagu (Western Cape) and Ectyphus armipes from Stellabush (KwaZulu-Natal), as well as the subspecies Ectyphus armipes pretoriensis from Pretoria (Gauteng). Hesse (1969) re-described Ectyphus with the addition of three new South African species all from the Eastern Cape: Ectyphus bitaeniatus from Resolution, Ectyphus capillatus from Brakkloof*,* and Ectyphus flavidorsalis from Willowmore. Also described were three varieties of Ectyphus pinguis: *litoralis* from the coastal Eastern Cape, and *ceramiiformis* and *karooensis* from Willowmore and surroundings. Hesse (1969) established Ectyphus armipes pretoriensis as a distinct species Ectyphus pretoriensis. Interestingly, Ectyphus capillatus was described only from two male specimens, Ectyphus flavidorsalis from two female specimens, and Ectyphus bitaeniatus from a single female. The status of the species and varieties described by Hesse (1969) is discussed below.

In 1972, Hesse described a new genus, Parectyphus from a single male specimen collected in Gobabeb, Erongo, Namibia. Illustrations of the antennae and hypopygium of the type species Parectyphus namibiensis Hesse, 1972 were provided. He postulated that Parectyphus was closely related to Ectyphus based on similar morphology, with one major difference being the elongation of the ‚stump vein‘ extending from wing vein R_4_ to connect with R_2+3_. Bowden (1980) catalogued 7 species for Ectyphus, Ectyphus abdominalis, Ectyphus armipes, Ectyphus bitaeniatus, Ectyphus capillatus, Ectyphus flavidorsalis, Ectyphus pinguis, and Ectyphus pretoriensis, and 1 species for Parectyphus, Parectyphus namibiensis.

Based on morphological similarity among Ectyphus, Opomydas, and the North American genus Heteromydas, Wilcox and Papavero (1971) erected the subfamily Ectyphinae. The taxon was based primarily on characters of the male terminalia: a free hypandrium, an aedeagus with a single tube, and ‚dististyli‘ on the gonocoxites. The term ‚dististyli‘ suggests that these novel appendages are gonostyli, which are absent in Mydidae (van Emden and Hennig 1970, Yeates and Irwin 1996, Dikow 2009). We, therefore, propose the term palp-like lateral appendage *sensu* Hesse (1969) to replace this term. Other characters diagnostic of Ectyphinae are the presence of macrosetae on the median surface of the metathoracic trochanters and metathoracic tarsomere 1 about five times as long as broad. Wilcox and Papavero (1971) list the Oriental Region along with the south-western USA, northern Mexico, and southern Africa as being inhabited by Ectyphinae, but later (Papavero and Wilcox 1974) the authors did not include this particular record from Asia. The genus Parectyphus was classified as *incertae sedis* by Papavero and Wilcox (1974) in their comprehensive study of the world Mydidae, but Bowden (1980), in the Catalogue of the Diptera of the Afrotropical Region, placed it within Ectyphinae.

## Materials and methods

Morphological terminology follows the Manual of Nearctic Diptera (McAlpine 1981) and Dikow (2009). Abdominal tergites and sternites are referred to as ‚T‘ and ‚S‘ respectively. The terms prothoracic, mesothoracic, and metathoracic are abbreviated ‘pro’, ‘mes’, and ‘met’, respectively. The term pubescence (adjective ‚pubescent‘) refers to the short, fine microtrichia densely covering certain body parts. Other generalised terms refer to the Torre-Bueno Glossary of Entomology (Nichols 1989).

Species descriptions are based on all available specimens. Well-preserved specimens exhibiting intraspecific variation were selected for description. The descriptions are compiled from a character matrix of 145 features assembled with Lucid Builder (v.3.5) and exported as natural language descriptions. When available, species are fully described in the male sex while females are only described with those features that differ (except for characters relating to the terminalia/genitalia). All specimens examined were dry-mounted on pins. Regarding the specimens selected for dissection, the female genitalia and male terminalia were excised and macerated in 10% potassium hydroxide at 55°C and rinsed in distilled H_2_O. The terminalia were stored in 70% ethanol for examination and illustration, but permanently stored in 100% glycerine. Morphological features were illustrated using a *camera lucida* on a Leica stereo-microscope and digitally re-drawn in Adobe Illustrator®. The vestiture/setation of the male terminalia was not illustrated. Wing length was measured from the tegula to the apex of the wing. Photographs of the specimens were taken using a Microptics ML Macro XLT digital system with a Canon EOS 40D camera. All photographs were deposited in Morphbank (http://www.morphbank.net) and permanent links to the full-size images are included in the figure captions.

In recording data for type specimens as well as non-type specimens, information is given (where available) in a standard manner, *i.e.*, locality, geographic co-ordinates, elevation, date of collection (month indicated in lower case Roman numerals where hyphens indicate missing entries for day, month, year), habitat information, collector, and depository. Female (♀) and male (♂) symbols indicate the sex while a question mark (?) refers to specimens of indeterminable sex (*i.e.*, with broken or missing abdomen). Each specimen (other than type specimens of already described species, which are sufficiently identified by their type status), is listed with a unique AAM specimen number that is attached as a white label and will allow the re-investigation as well as provide a unique identifier (LSID http://lsids.sourceforge.net) in databases like GBIF (http://www.gbif.org) in the future. AAM is an abbreviation for ‘Apioceridae Asilidae Mydidae’ and identifies a record in the specimen database used by T. Dikow in this format: AAM-000000. The distribution of all studied specimens is illustrated in distribution maps created in ArcMap (v.9). The electronic shape-files of the Biodiversity Hotspots were obtained from Conservation International (2005). The electronic keys were deposited in the IdentifyLife (http://www.identifylife.org) project.

Institutions providing specimens are listed below, along with the abbreviations used in the text and the people who kindly assisted: AMGS - Albany Museum, Grahamstown, Eastern Cape, South Africa (A. Kirk-Spriggs, S. Gess); BMNH - The Natural History Museum, London, UK (E. McAlister,); CAS - California Academy of Sciences, San Francisco, California, USA (C. Griswold); CNC - Canadian National Collection of Insects, Arachnids and Nematodes, Ottawa, Ontario, Canada (J. Skevington); DEIC - Senckenberg Deutsches Entomologisches Institut, Müncheberg, Brandenburg, Germany (F. Menzel); ISNB - Institut Royal des Sciences Naturelles de Belgique, Brussels, Belgium (P. Grootaert); MNHN - Museum national d’Histoire naturelle, Paris, France (C. Daugeron, E. Delfosse); MZLU - Museum of Zoology, Lund University, Lund, Sweden (R. Danielsson); NMNW - National Museum of Namibia, Windhoek, Namibia (A. Kirk-Spriggs); NMSA - Natal Museum, Pietermaritzburg, KwaZulu-Natal, South Africa (B. Muller, M. Mostovski); SAMC - South African Museum, Cape Town, Western Cape, South Africa (M. Cochrane); SANC - South African National Collection of Insects, Pretoria, Gauteng, South Africa (R. Urban); SMNS - Staatliches Museum für Naturkunde, Stuttgart, Baden-Württemberg, Germany (H.-P. Tschorsnig); USNM - United States National Museum, Smithsonian Institution, Washington, DC, USA (F.C. Thompson); ZMHB - Museum für Naturkunde, Berlin, Germany (J. Ziegler, J. Pohl); ZSMC - Zoologische Staatssammlung, München, Bayern, Germany (M. Kotrba).

## Taxonomy

### Genus Ectyphus

Gerstaecker, 1868

Ectyphus Gerstaecker 1868: 92. Type species: Ectyphus pinguisGerstaecker 1868, by monotypy.

#### Diagnosis:

Ectyphus is distinguished from other Afrotropical Mydidae by the distinctly clubbed metathoracic femur, the presence of a ventral keel on the metathoracic tibia terminating into a well-developed apical spine, and veins M_3_+CuA_1_ terminate together into C on the posterior wing margin. Other features include the presence of 3 spermathecae in females and a free, square, and more or less flat hypandrium in males.

### Ectyphus abdominalis

Bezzi, 1924

Figs 2 45

Ectyphus abdominalis Bezzi 1924: 198; Hesse 1969: 378; Bowden 1980: 326.

#### Diagnosis:

**Figures 2–5.**
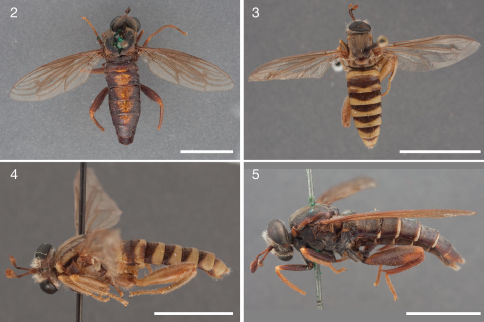
Photographs of Ectyphus species. **2** Ectyphus abdominalis (♀ holotype, SAMC, Morphbank); **3–4** Ectyphus amboseli sp. n. (♂ holotype, AAM-000191, CAS) **3** dorsal (Morphbank) **4** lateral (Morphbank) **5** Ectyphus armipes (♂ holotype, SAMC, Morphbank). Scale lines = 5 mm.

The species is distinguished from congeners by the broad, reddish stripe covering most of the dorsal abdomen (Fig. 2), the light brown setation on the head and scutum, the lack of a yellow posterior margin on the abdominal tergites, and its apparent distribution in the western Western Cape Province.

#### Re-description female:

##### Head:

brown, facial gibbosity yellow, in general white pubescent; width distinctly greater than thorax, interocular distance on vertex larger than at ventral eye margin, vertex between compound eyes slightly depressed, parafacial area very narrow, facial gibbosity nearly touching median eye margin; facial gibbosity distinct, well-developed and discernible in lateral view; mystax light brown, covering entire facial gibbosity; frons predominantly white pubescent (only narrow median area apubescent), vertex entirely white pubescent, postgena white pubescent; setation: vertex light brown, frons light brown, ocp setae brown, pocl setae brown; ocellar triangle apubescent; proboscis brown, short, about ½ length of oral cavity; labellum small, as wide as prementum, about ½ length of prementum, unsclerotised laterally; maxillary palpus cylindrical, light brown, longer than ½ length of proboscis.

##### Antenna:

brown, scape and pedicel brown setose dorsally and ventrally; postpedicel cylindrical in proximal ½, symmetrically bulbous in distal ½, ≥ 5.0 times as long as combined length of scape and pedicel; apical ‚seta-like‘ sensory element situated apically in cavity on postpedicel.

##### Thorax:

brown, predominantly yellow pubescent; scutum medially bluish-black, laterally brown, surface entirely smooth, lightly grey pubescent, scutal setation comprised of distinct rows of short dorsocentral setae and lateral scutal setae; dc setae pre- and postsuturally light brown, acr setae absent, lateral scutal setae brown, npl, spal, and pal setae absent; postpronotal lobe light brown, partly silver pubescent; proepisternum, lateral postpronotum, and postpronotal lobe long brown setose; scutellum entirely silver pubescent, short brown setose, apical scutellar setae absent; mesopostnotum, anatergite, and katatergite grey pubescent, asetose; katatergite elevated and smoothly convex; anterior anepisternum asetose, supero-posterior anepisternum asetose; posterior anepimeron long white setose, katepimeron asetose; metepimeron evenly elevated, same colour as T1, silver pubescent, asetose; metepisternum silver pubescent, asetose.

##### Leg:

brown, setation predominantly white; pro, mes, and met coxa grey pubescent, white setose; met trochanter macrosetose medially; femur brown, met femur evenly clubbed in distal ¾, in distal ½ macrosetose, 1 antero-ventral and 1 postero-ventral row of macrosetae; pro, mes, and met tibia straight, met tibia cylindrical with distinct ventral keel terminating into a sharp spine; pro and mes tarsomere 1 as long as combined length of tarsomeres 2–3, pulvillus well-developed, as long as well-developed claw, and as wide as base of claw; empodium absent.

##### Wing:

length = 13.2 mm; hyaline throughout, slightly brown stained along veins, veins light brown, microtrichia absent; cells r_1_, r_4_, r_5_, m_3_, + cu*p* closed; C well-developed, around entire wing; R_4_ terminates in R_1_; R_5_ terminates in R_1_; stump vein (R_3_) at base of R_4_ present, long but not reaching R_2_; R_4_ and R_5_ widest apart medially; r-m distinct, R_4+5_+5 and M_1_ apart, connected by crossvein; M_1_ straight at r-m (not curving anteriorly), M_1_ (or M_1_+M_2_) terminates in C; CuA_1_ and CuA_2_ split proximally to m-cu (cell m_3_ narrow proximally); M_3_+CuA_1_ terminate together in C; A_1_ undulating, cell a_1_ wide, A_1_ and wing margin further apart proximally than distally; alula well-developed; halter brown.

##### Abdomen:

brown and yellow; setation comprised of sparsely scattered short brown setae, surface entirely smooth; T1 brown, T2 brown with yellow anterior and posterior margin, T3–7 brown laterally and yellow medially; T1–3 sparsely brown setose; T predominantly apubescent; S1–7 light brown; S1–3 asetose; S predominantly apubescent; T2–4 parallel-sided and not constricted waist-like; bullae on T2 black, transversely elongate, surface entirely smooth, T2 surface anterior to bullae smooth.

##### Female genitalia:

densely arranged anteriorly directed setae absent, only few on T7–8 and S7–8; T10 divided into 2 heavily sclerotised acanthophorite plates. Specimen not further dissected to preserve the unique, already damaged holotype.

#### Re-description male:

male unknown.

#### Material examined:

**South Afric**a: Western Cape Province: 1♀ Montagu, 33°47'12"S; 20°06'42"E, -.i.1876, R. Turner (holotype, SAMC).

#### Type locality, distribution, and biodiversity hotspot:

Montagu (33°47'12"S; 20°06'42"E), Western Cape, South Africa. Cape Floristic Region biodiversity hotspot.

#### Remarks:

Although we were able to study some 131 specimens of Ectyphus, many of them new records since the last review by Hesse (1969), we were unable to identify any additional specimen of Ectyphus abdominalis. The unique female holotype is in poor condition and originates from a unique locality from where no other Ectyphus have ever been collected (Fig. 45). It is possible that this species represents another junior synonym of Ectyphus pinguis as this species, which is primarily known from the Eastern Cape, occurs even further west in the Western Cape than the type locality of Ectyphus abdominalis (see Remarks under Ectyphus pinguis). Only when male specimens from the type locality become available can the status of this species be confirmed.

### Ectyphus amboseli sp. n.

urn:lsid:zoobank.org:act:482F4960-9312-4110-ADF2-51402FC2642F

Figs 1 3–4 11–13 

#### Etymology:

Noun in apposition that refers to the type locality Amboseli Lodge, Kenya.

#### Diagnosis:

The species is distinguished from congeners by the yellow colour and pubescence of the thorax (Fig. 3), the yellow abdominal sternites (Fig. 3), and its apparent distribution in Kenya (Fig. 1).

#### Description male:

##### Head:

brown, facial gibbosity yellow, in general grey pubescent; width distinctly greater than thorax, interocular distance on vertex larger than at ventral eye margin, vertex between compound eyes slightly depressed, parafacial area very narrow, facial gibbosity nearly touching median eye margin; facial gibbosity distinct, well-developed and discernible in lateral view; mystax white, covering entire facial gibbosity; frons entirely grey pubescent, vertex medially apubescent, laterally grey pubescent, postgena white pubescent; setation: vertex white, frons white, ocp setae white, pocl setae white; ocellar triangle apubescent; proboscis brown, short, about ½ length of oral cavity; labellum small, as wide as prementum, as long as prementum, unsclerotised laterally; maxillary palpus cylindrical, light brown, about ½ length of proboscis.

##### Antenna

brown, scape and pedicel white setose dorsally and ventrally; postpedicel cylindrical in proximal ½, symmetrically bulbous in distal ½, ≥ 6.0 times as long as combined length of scape and pedicel; apical ‚seta-like‘ sensory element situated apically in cavity on postpedicel.

##### Thorax:

yellow, predominantly yellow pubescent; scutum yellow, broad brown median presutural stripe and brown paramedial postsutural stripes, surface entirely smooth, predominantly yellow pubescent, paramedial and sublateral stripes apubescent, scutal setation comprised of distinct rows of short dorsocentral setae and lateral scutal setae; dc setae pre- and postsuturally white, acr setae absent, lateral scutal setae white, npl, spal, and pal setae absent; postpronotal lobe yellow, partly white pubescent; proepisternum, lateral postpronotum, and postpronotal lobe long white setose; scutellum apubescent, asetose medially, laterally yellow setose, apical scutellar setae absent; mesopostnotum, anatergite, and katatergite silver pubescent, asetose; katatergite elevated and smoothly convex; anterior anepisternum asetose, supero-posterior anepisternum asetose; posterior anepimeron long white setose, katepimeron asetose; metepimeron evenly elevated, same colour as T1, silver pubescent, asetose; metepisternum silver pubescent, asetose.

##### Leg:

yellow, setation predominantly white; pro, mes, and met coxa grey pubescent, white setose; met trochanter macrosetose medially; femur yellow, met femur evenly clubbed in distal ¾, in distal ½ macrosetose, 1 antero-ventral and 1 postero-ventral row of macrosetae; pro, mes, and met tibia straight, met tibia cylindrical with distinct ventral keel terminating into a sharp spine; pro and mes tarsomere 1 longer than tarsomere 2, but less than combined length of tarsomeres 2–3, met tarsomere 1 as long as combined length of tarsomeres 2–3; pulvillus well-developed, as long as well-developed claw, and as wide as base of claw; empodium absent.

##### Wing:

length = 9.8–10.3 mm; hyaline throughout, veins light brown, microtrichia absent; cells r_1_, r_4_, r_5_, m_3_, + cu*p* closed; C well-developed, around entire wing; R_4_ terminates in R_1_; R_5_ terminates in R_1_; stump vein (R_3_) at base of R_4_ present, short not reaching R_2_; R_4_ and R_5_ widest apart medially; r-m distinct, R_4_+5 and M_1_ apart, connected by crossvein; M_1_ straight at r-m (not curving anteriorly), M_1_ (or M_1_+M_2_) terminates in C; CuA_1_ and CuA_2_ split proximally to m-cu (cell m_3_ narrow proximally); M_3_+CuA_1_ terminate together in C; A_1_ undulating, cell a_1_ wide, A_1_ and wing margin further apart proximally than distally; alula well-developed; halter light yellow.

##### Abdomen:

brown and yellow; setation comprised of scattered white setae, surface entirely smooth; T1–7 brown, yellow posterior margin; T1 long white setose, T2–T3 sparsely white setose; T predominantly apubescent; S1–7 yellow; S1 asetose, S2–3 sparsely white setose; S predominantly apubescent; T2–4 parallel-sided and not constricted waist-like; bullae on T2 black, transversely elongate, surface entirely smooth, T2 surface anterior to bullae smooth.

##### Male terminalia:

T1–7 well-developed, entirely sclerotised, T8 postero-medially weakly sclerotised, with anterior transverse sclerotised bridge connecting lateral sclerites; T7–8 anteriorly with 2 lateral apodemes; S6 regular, without any special setation postero-medially, S8 well-developed and simple, fused to T8 dorso-laterally, entire (undivided) ventro-medially; epandrium formed by single sclerite (fused medially ± entirely), pointed postero-laterally; subepandrial sclerite without lateral or median protuberances; hypandrium ± flat, rectangular to square sclerite, entirely fused with gonocoxite, forming a gonocoxite-hypandrial complex; gonocoxite dorso-ventrally flattened in distal ½, higher in proximal ½, with palp-like lateral appendage, gonocoxal apodeme present, short (at most slightly extending hypopygium anteriorly); 1 functional aedeagal prong, aedeagal epimere absent; lateral ejaculatory process absent; ejaculatory apodeme formed by single dorso-ventrally oriented plate; ventro-median margin of dorsal aedeagal sheath heavily sclerotised (appearing entirely closed); dorsal aedeagal sheath long, sperm sac entirely covered; sperm sac appearing ± heavily sclerotised.

#### Description female:

female unknown.

#### Material examined:

**Keny**a: Rift Valley Province: 3♂ 1? Amboseli Lodge, 2°39'59"S; 37°17'00"E, 28.ix.1972, W. Middlekauff (holotype AAM-000191, paratypes AAM-000190, AAM-000192–AAM-000193, CAS).

#### Type locality, distribution, and biodiversity hotspot:

Amboseli Lodge (2°39'59"S; 37°17'00"E), Kenya. Does not occur in any currently recognised biodiversity hotspot.

### Ectyphus armipes

Bezzi, 1924

Figs 5 6 14–16 45

Ectyphus armipes Bezzi 1924: 196; Hesse 1969: 381; Wilcox and Papavero 1971: 59; Bowden 1980: 326.

#### Diagnosis:

The species is distinguished from congeners by the long proboscis that is slightly longer than the oral cavity, the large labellum that occupies nearly the entire oral cavity, brown facial gibbosity and postpronotal lobe, and the dorso-ventrally flattened ‘palp-like’ appendage on the gonocoxite in males.

**Figures 6–10.**
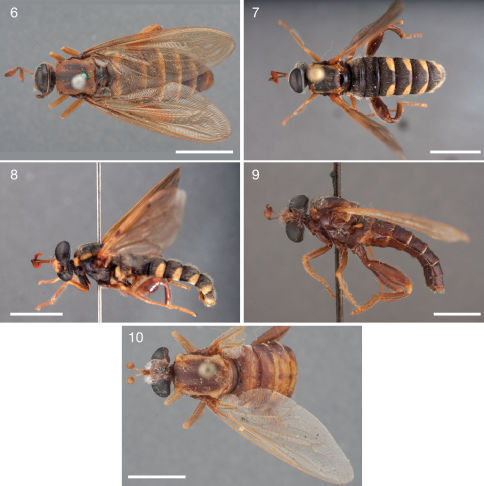
Photographs of Ectyphus species. **6** Ectyphus armipes (♀ paratype, SAMC, Morphbank); **7–8** Ectyphus capillatus (♂, AAM-003502, AMGS) **7** dorsal (Morphbank) **8** lateral (Morphbank) **9** Ectyphus pretoriensis (♂ lectotype, SAMC, Morphbank) **10** Ectyphus pretoriensis (♀ paralectotype, SAMC, Morphbank). Scale lines = 5 mm.

**Figures 11–19.**
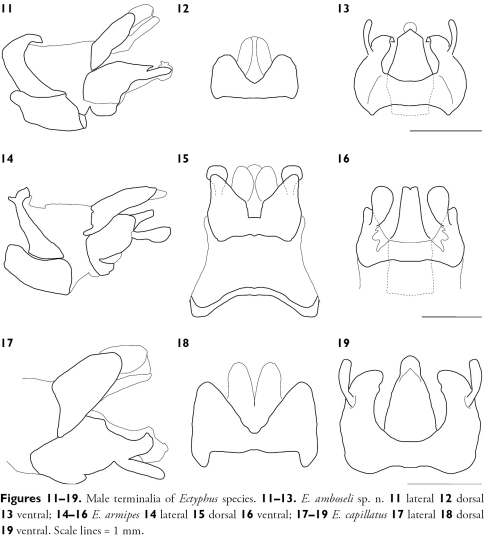
Male terminalia of Ectyphus species. **11–13.** Ectyphus amboseli sp. n. **11** lateral **12** dorsal **13** ventral; **14–16** Ectyphus armipes **14** lateral **15** dorsal **16** ventral; **17–19** Ectyphus capillatus **17** lateral **18** dorsal **19** ventral. Scale lines = 1 mm.

#### Re-description male:

##### Head:

black, facial gibbosity light brown, in general predominantly apubescent, yellow pubescent on median eye margin; width distinctly greater than thorax, interocular distance on vertex larger than at ventral eye margin, vertex between compound eyes slightly depressed, parafacial area very narrow, facial gibbosity nearly touching median eye margin; facial gibbosity distinct, well-developed and discernible in lateral view; mystax white, covering entire facial gibbosity, sparse; frons predominantly apubescent, vertex apubescent, postgena lightly silver pubescent; setation: vertex white, frons white, ocp setae white, pocl setae white; ocellar triangle apubescent; proboscis light brown, long, reaching fronto-clypeal suture; labellum large, much wider than prementum, longer than prementum and as long as oral cavity, unsclerotised laterally; maxillary palpus cylindrical, light brown, about ½ length of proboscis.

##### Antenna

brown, scape and pedicel white and yellow setose dorsally and ventrally; postpedicel cylindrical in proximal ½, symmetrically bulbous in distal ½, ≥ 6.0 times as long as combined length of scape and pedicel; apical ‚seta-like‘ sensory element situated apically in cavity on postpedicel.

##### Thorax:

dark brown to bluish-black, predominantly apubescent; scutum medially dark brown, laterally brown, surface entirely smooth, predominantly apubescent, only extreme lateral margin grey pubescent, scutal setation comprised of distinct rows of short dorsocentral setae and lateral scutal setae; dc setae pre- and postsuturally white, acr setae absent, lateral scutal setae white, npl, spal, and pal setae absent; postpronotal lobe light brown, partly silver pubescent; proepisternum, lateral postpronotum, and postpronotal lobe long white setose; scutellum apubescent, asetose medially, laterally yellow setose, apical scutellar setae absent; mesopostnotum, anatergite, and katatergite silver pubescent, asetose; katatergite elevated and smoothly convex; anterior anepisternum asetose, supero-posterior anepisternum asetose; posterior anepimeron long white setose, katepimeron asetose; metepimeron evenly elevated, same colour as T1, silver pubescent, asetose; metepisternum silver pubescent, asetose.

##### Leg:

brown, setation predominantly white; pro, mes, and met coxa apubescent, long white setose; met trochanter macrosetose medially; femur brown, met femur evenly clubbed in distal ¾, in distal ½ macrosetose, 1 antero-ventral and 1 postero-ventral row of macrosetae; pro, mes, and met tibia straight, met tibia cylindrical with distinct ventral keel terminating into a sharp spine; pro and mes tarsomere 1 as long as combined length of tarsomeres 2–3, met tarsomere 1 as long as combined length of tarsomeres 2–4; pulvillus well-developed, as long as well-developed claw, and as wide as base of claw; empodium absent.

##### Wing:

length = 12.2–14.1 mm; slightly brown stained, darker brown around veins, veins light brown, microtrichia absent; cells r_1_, r_4_, r_5_, m_3_, + cu*p* closed; C well-developed, around entire wing; R_4_ terminates in R_1_; R_5_ terminates in R_1_; stump vein (R_3_) at base of R_4_ present, long but not reaching R_2_; R_4_ and R_5_ widest apart medially; r-m distinct, R_4_+5 and M_1_ apart, connected by crossvein; M_1_ straight at r-m (not curving anteriorly), M_1_ (or M_1_+M_2_) terminates in C; CuA_1_ and CuA_2_ split proximally to m-cu (cell m_3_ narrow proximally); M_3_+CuA_1_ terminate together in C; A_1_ undulating, cell a_1_ wide, A_1_ and wing margin further apart proximally than distally; alula well-developed; halter light brown.

##### Abdomen:

brown; setation comprised of scattered white setae, surface entirely smooth; T1 brown, T2–7 brown with yellow posterior margin; T1 long white setose, T2–T3 sparsely white setose; T predominantly apubescent; S1–7 brown, yellow posterior margin; S1 asetose, S2–3 sparsely white setose; S predominantly apubescent; T2–4 parallel-sided and not constricted waist-like; bullae on T2 brown, transversely elongate, surface entirely smooth, T2 surface anterior to bullae smooth.

##### Male terminalia:

T1–7 well-developed, entirely sclerotised, T8 postero-medially weakly sclerotised, with anterior transverse sclerotised bridge connecting lateral sclerites; T7–8 anteriorly with 2 lateral apodemes; S6 regular, without any special setation postero-medially, S8 well-developed and simple, fused to T8 dorso-laterally, entire (undivided) ventro-medially; epandrium formed by single sclerite (fused medially ± entirely), pointed postero-laterally; subepandrial sclerite without lateral or median protuberances; hypandrium ± flat, rectangular to square sclerite, entirely fused with gonocoxite, forming a gonocoxite-hypandrial complex; gonocoxite dorso-ventrally flattened in distal ½, higher in proximal ½, with palp-like lateral appendage, gonocoxal apodeme present, short (at most slightly extending hypopygium anteriorly); 1 functional aedeagal prong, aedeagal epimere absent; lateral ejaculatory process absent; ejaculatory apodeme formed by single dorso-ventrally oriented plate; ventro-median margin of dorsal aedeagal sheath heavily sclerotised (appearing entirely closed); dorsal aedeagal sheath long, sperm sac entirely covered; sperm sac appearing ± heavily sclerotised.

#### Re-description female:

##### Head:

proboscis brown; maxillary palpus brown.

##### Antenna

postpedicel ≥ 5.0 times as long as combined length of scape and pedicel.

##### Thorax:

light brown, predominantly grey pubescent; scutum yellow, broad brown median presutural stripe and brown paramedial postsutural stripes; scutum lightly grey pubescent.

##### Leg:

yellow; pro, mes, and met coxa grey pubescent, white setose; femur yellow.

##### Wing:

length = 14.1–14.5 mm; hyaline throughout, slightly brown stained along veins.

##### Abdomen:

T1–7 brown, yellow posterior margin; T1–3 sparsely white setose; S1–7 brown; bullae on T2 black, transversely elongate.

##### Female genitalia:

densely arranged anteriorly directed setae absent, only few on T7–8 and S7–8; T8 with broad anterior rectangular apodeme; T9 formed by wide, rectangular sclerite with median protuberance; T9+10 entirely fused, T10 divided into 2 heavily sclerotised acanthophorite plates, 11 acanthophorite spurs per plate; 3 spermathecae, all equally large, formed by ± expanded weakly sclerotised ducts; individual spermathecal duct long; S9 (furca) formed by 2 sclerites, separated anteriorly and posteriorly, anterior furcal apodeme present, 2 lateral projections forming divided apodeme, lateral furcal apodeme absent, median furcal bridge absent.

#### Material examined:

**South Africa:** KwaZulu-Natal: 1♂ St. Lucia Lake, 27°56'43"S; 32°26'11"E, 4.xi.1920, H. Bell Marley (AAM-003454, NMSA); 1♀ 1♂ Mtunzini, 28°57'00"S; 31°45'00"E, 1.xii.1980, R. Oberprieler (AAM-003490–AAM-003491, SANC); 2♂ Tongaat, 29°34'12"S; 31°07'06"E, -.ix.1908, H. Burnup (AAM-003452–AAM-003453, NMSA); 1♂ Tongaat, -.-.1908–1909, H. Burnup (AAM-003476, BMNH); 1♀ 1♂ Tongaat River, 29°34'13"S; 31°10'47"E, -.-.1908–1909, H. Burnup (AAM-003474–AAM-003475, BMNH); 2♀ Stellabush (= Pigeon Valley Nature Reserve), 29°51'00"S; 30°59'00"E, -.-.1915, H. Bell Marley (AAM-003457–AAM-003458, NMSA); 1♀ 1♂ Stellabush (= Pigeon Valley Nature Reserve), -.i.1915, H. Bell Marley (holotype and paratype, SAMC); 1♀ Stellabush (= Pigeon Valley Nature Reserve), -.-.1915, H. Bell Marley (AAM-003511, SAMC); 5♂ Durban, 29°51'00"S; 31°01'00"E, -.i–iii.1959, C. Booth (AAM-003513–AAM-003517, SAMC); 1♀ 1♂ Durban, 13.ii.1963, G. Heinrich (AAM-003483–AAM-003484, CNC); 1? Natal (= Durban), -.iv.1868, W. Saunders (AAM-003477, BMNH); 1♂ Natal (= Durban), -.-.1904, J. Gregoe (AAM-003480, BMNH); 1♂ Natal (= Durban), -.vii.1942, (AAM-003488, SAMC); 1♀ Port-Natal (= Durban), -.-.-, Plant (AAM-003479, BMNH); 1♂ Bluff, Durban, 29°53'00"S; 31°03'00"E, 18.i.1904, C. Barker (AAM-003456, NMSA); 1♂ Amanzimtoti, 30°03'00"S; 30°53'00"E, -.i.1950, (AAM-003455, NMSA); 1♀ Widenham, 30°12'57"S; 30°47'47"E, 2.i.1915, E. Chubb (AAM-003481, BMNH); 1♂ Widenham, 20.xii.1914, (AAM-003512, SAMC); **No locality information**: 1♂, Plant (AAM-003478, BMNH); 1♀ (AAM-003482, BMNH).

#### Type locality, distribution, and biodiversity hotspot:

Stellabush (now Pigeon Valley Nature Reserve, 29°51'51"S; 30°59'13"E), Durban, KwaZulu-Natal, South Africa. Maputaland-Pondoland-Albany biodiversity hotspot.

#### Remarks:

Three specimens, two from Tongaat River (AAM-003474–AAM-003475) and one from Tongaat (AAM-003476), all collected in 1908–1909 exhibit a long stump vein (R_3_) entirely connecting veins R_2_ and R_4_ (*e.g.*, Fig. 38). The presence of this connecting stump vein is otherwise only known from and diagnostic for the genus Parectyphus (see below). Ectyphus armipes, however, does not show any of the other diagnostic features of Parectyphus and the male terminalia exhibit the usual Ectyphus configuration so that we view the presence of this stump vein as a morphological anomaly.

### Ectyphus capillatus

Hesse, 1969

Figs 7–8 17–19 45

Ectyphus capillatus Hesse 1969: 376; Bowden 1980: 326.

#### Diagnosis:

The species is distinguished from congeners by the yellow facial gibbosity, the distinctly yellow metepimeron, and the dense and long white setae on abdominal tergites 5–7.

#### Re-description male:

##### Head:

black, facial gibbosity yellow, in general lightly silver pubescent; width distinctly greater than thorax, interocular distance on vertex larger than at ventral eye margin, vertex between compound eyes slightly depressed, parafacial area very narrow, facial gibbosity nearly touching median eye margin; facial gibbosity distinct, well-developed and discernible in lateral view; mystax white, covering only lateral facial gibbosity (asetose medially); frons medially apubescent, laterally grey pubescent, vertex predominantly apubescent, only lateral margin grey pubescent, postgena lightly silver pubescent; setation: vertex white, frons white, ocp setae white, pocl setae white; ocellar triangle apubescent; proboscis brown, short, about ½ length of oral cavity; labellum small, as wide as prementum, as long as prementum, unsclerotised laterally; maxillary palpus cylindrical, brown, longer than ½ length of proboscis.

##### Antenna

brown, scape and pedicel white and yellow setose dorsally and ventrally; postpedicel cylindrical in proximal ½, symmetrically bulbous in distal ½, ≥ 7.0 times as long as combined length of scape and pedicel; apical ‚seta-like‘ sensory element situated apically in cavity on postpedicel.

##### Thorax:

brown, lightly grey pubescent; scutum yellow, broad brown median presutural stripe and brown paramedial postsutural stripes, surface entirely smooth, predominantly yellow pubescent, paramedial and sublateral stripes apubescent, scutal setation comprised of distinct rows of long dorsocentral setae and lateral scutal setae; dc setae pre- and postsuturally light brown, acr setae absent, lateral scutal setae white, npl, spal, and pal setae absent; postpronotal lobe yellow, partly white pubescent; proepisternum, lateral postpronotum, and postpronotal lobe long white setose; scutellum apubescent, asetose, apical scutellar setae absent; mesopostnotum, anatergite, and katatergite grey pubescent, asetose; katatergite elevated and smoothly convex; anterior anepisternum asetose, supero-posterior anepisternum asetose; posterior anepimeron long white setose, katepimeron asetose; metepimeron evenly elevated, yellow, lightly silver pubescent, asetose; metepisternum silver pubescent, asetose.

##### Leg:

light brown, setation predominantly white; pro, mes, and met coxa apubescent, long white setose; met trochanter macrosetose medially; femur light brown, met femur evenly clubbed in distal ¾, in distal ½ macrosetose, 1 antero-ventral and 1 postero-ventral row of macrosetae; pro, mes, and met tibia straight, met tibia cylindrical with distinct ventral keel terminating into a sharp spine; pro and mes tarsomere 1 longer than tarsomere 2, but less than combined length of tarsomeres 2–3, met tarsomere 1 as long as combined length of tarsomeres 2–3; pulvillus well-developed, as long as well-developed claw, and as wide as base of claw; empodium absent.

##### Wing:

length = 10.7–13.1 mm; hyaline throughout, slightly brown stained along veins, veins light brown, microtrichia absent; cells r_1_, r_4_, r_5_, m_3_, + cu*p* closed; C well-developed, around entire wing; R_4_ terminates in R_1_; R_5_ terminates in R_1_; stump vein (R_3_) at base of R_4_ present, long but not reaching R_2_; R_4_ and R_5_ widest apart medially; r-m distinct, R_4_+5 and M_1_ apart, connected by crossvein; M_1_ straight at r-m (not curving anteriorly), M_1_ (or M_1_+M_2_) terminates in C; CuA_1_ and CuA_2_ split proximally to m-cu (cell m_3_ narrow proximally); M_3_+CuA_1_ terminate together in C; A_1_ undulating, cell a_1_ wide, A_1_ and wing margin further apart proximally than distally; alula well-developed; halter light yellow.

##### Abdomen:

brown; setation comprised of dense long white setose, surface microrugose; T1 brown, T2–7 brown with yellow posterior margin broadly interrupted medially; T1–3 densely long white setose; T entirely grey pubescent; S1 light brown, S2–5 yellow, brown anteriorly, S6–7 brown with yellow posterior margin; S1 asetose, S2–3 sparsely white setose; S predominantly apubescent; T2–4 parallel-sided and not constricted waist-like; bullae on T2 black, transversely elongate, surface entirely smooth, T2 surface anterior to bullae smooth.

##### Male terminalia:

T1–7 well-developed, entirely sclerotised, T8 postero-medially weakly sclerotised, with anterior transverse sclerotised bridge connecting lateral sclerites; T7–8 anteriorly with 2 lateral apodemes; S6 regular, without any special setation postero-medially, S8 well-developed and simple, fused to T8 dorso-laterally, entire (undivided) ventro-medially; epandrium formed by single sclerite (fused medially ± entirely), rounded postero-laterally; subepandrial sclerite without lateral or median protuberances; hypandrium ± flat, rectangular to square sclerite, entirely fused with gonocoxite, forming a gonocoxite-hypandrial complex; gonocoxite dorso-ventrally flattened in distal ½, higher in proximal ½, with palp-like lateral appendage, gonocoxal apodeme present, short (at most slightly extending hypopygium anteriorly); 1 functional aedeagal prong, aedeagal epimere absent; lateral ejaculatory process absent; ejaculatory apodeme formed by single dorso-ventrally oriented plate; ventro-median margin of dorsal aedeagal sheath heavily sclerotised (appearing entirely closed); dorsal aedeagal sheath long, sperm sac entirely covered; sperm sac appearing ± heavily sclerotised.

#### Description female:

female unknown.

#### Material examined:

**South Africa:** Eastern Cape Province: 1♂ Double Drift, Andries Vosloo Kudu Reserve, 33°06'00"S; 26°47'00"E, 14.xii.1988, A. Weaving (AAM-003502, AMGS); 1♂ Resolution, 33°10'00"S; 26°37'00"E, 4.i.1928, A. Walton (paratype, NMSA); 1♂ Brakkloof, 33°12'00"S; 26°50'00"E, -.-.1907, G. White (holotype, SAMC).

#### Type locality, distribution, and biodiversity hotspot:

Brakkloof (33°12'00"S; 026°50'00"E), Eastern Cape, South Africa. Maputaland-Pondoland-Albany biodiversity hotspot.

#### Remarks:

The ♂ paratype specimen was collected at Resolution, which is also the type locality of Ectyphus bitaeniatus Hesse, 1969 (synonymised with Ectyphus pinguis below). Ectyphus bitaeniatus is known from the ♀ holotype only. Although both specimens from Resolution were collected by the same collector (A. Walton), the specimens originate from separate collecting events although during the same summer of 1927–1928 and were collected only some 12 days apart. Hesse (1969: 377) mistakenly lists the Ectyphus capillatus paratype to be collected in January 1924 while the label indicates January 1928 (B. Muller pers. comm.). As Ectyphus capillatus is still only known in the ♂ sex and no other species of Ectyphus has ever been collected at Resolution, it is possible that Ectyphus bitaeniatus represents the ♀ of Ectyphus capillatus. Until more specimens from this area north-east of Grahamstown, in which all three collecting localities of Ectyphus capillatus are situated, become available we cannot definitely provide confirmation of the possible synonymy. Ectyphus capillatus would take priority by page number.

### Ectyphus pinguis

Gerstaecker, 1868

**Figures 20–30.**
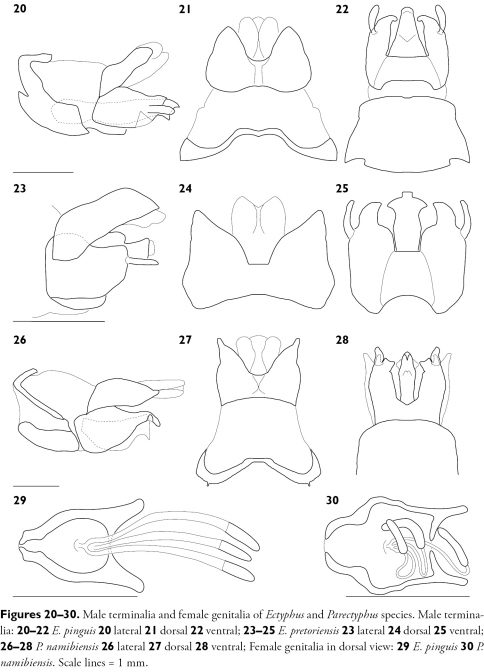
Male terminalia and female genitalia of Ectyphus and Parectyphus species. Male terminalia: **20–22** Ectyphus pinguis **20** lateral **21** dorsal **22** ventral; **23–25** Ectyphus pretoriensis **23** lateral **24** dorsal **25** ventral; **26–28** Parectyphus namibiensis **26** lateral **27** dorsal **28** ventral; Female genitalia in dorsal view: **29** Ectyphus pinguis **30** Parectyphus namibiensis. Scale lines = 1 mm.

Figs 20–22, 29 31 37 45

Ectyphus pinguis Gerstaecker 1868: 92; Bezzi 1924: 196; Hesse 1969: 369; Bowden 1980: 326.
Ectyphus pinguis  var. litoralis Hesse 1969: 372. unavailable name Ectyphus pinguis var. karooensis Hesse 1969: 374. unavailable name Ectyphus pinguis var. ceramiiformis Hesse 1969: 375. unavailable name Ectyphus bitaeniatusHesse 1969: 380. syn. n. Ectyphus flavidorsalisHesse 1969: 378. syn. n.

#### Diagnosis:

The species is distinguished from congeners by the enlarged yellow facial gibbosity, the yellow posterior margin of the abdominal tergites that are widened laterally and interrupted medially, and the distinctly yellow metepimeron.

#### Re-description male:

##### Head:

black, facial gibbosity yellow, in general lightly silver pubescent; width distinctly greater than thorax, interocular distance on vertex larger than at ventral eye margin, vertex between compound eyes slightly depressed, parafacial area very narrow, facial gibbosity nearly touching median eye margin; facial gibbosity distinct, well-developed and discernible in lateral view; mystax white, covering only lateral facial gibbosity (asetose medially); frons medially apubescent, laterally grey pubescent, vertex apubescent, postgena lightly silver pubescent; setation: vertex white, frons white, ocp setae white, pocl setae white; ocellar triangle apubescent; proboscis light brown, short, about ½ length of oral cavity; labellum small, as wide as prementum, as long as prementum, unsclerotised laterally; maxillary palpus cylindrical, brown, longer than ½ length of proboscis.

##### Antenna

brown, scape and pedicel white and yellow setose dorsally and ventrally; postpedicel cylindrical in proximal ½, symmetrically bulbous in distal ½, ≥ 8.0 times as long as combined length of scape and pedicel; apical ‚seta-like‘ sensory element situated apically in cavity on postpedicel.

##### Thorax:

dark brown to bluish-black, predominantly grey pubescent; scutum medially brown, laterally dark yellow, surface entirely smooth, lightly grey pubescent, scutal setation comprised of distinct rows of short dorsocentral setae and lateral scutal setae; dc setae pre- and postsuturally white, acr setae absent, lateral scutal setae white, npl, spal, and pal setae absent; postpronotal lobe yellow, partly white pubescent; proepisternum, lateral postpronotum, and postpronotal lobe long white setose; scutellum apubescent, asetose medially, laterally yellow setose, apical scutellar setae absent; mesopostnotum, anatergite, and katatergite silver pubescent, asetose; katatergite elevated and smoothly convex; anterior anepisternum asetose, supero-posterior anepisternum asetose; posterior anepimeron long white setose, katepimeron asetose; metepimeron evenly elevated, yellow, lightly silver pubescent, asetose; metepisternum silver pubescent, asetose.

##### Leg:

light brown, setation predominantly white; pro, mes, and met coxa apubescent, long white setose; met trochanter macrosetose medially; femur brown, met femur evenly clubbed in distal ¾, in distal ½ macrosetose, 1 antero-ventral and 1 postero-ventral row of macrosetae; pro, mes, and met tibia straight, met tibia cylindrical with distinct ventral keel terminating into a sharp spine; pro and mes tarsomere 1 longer than tarsomere 2, but less than combined length of tarsomeres 2–3, met tarsomere 1 as long as combined length of tarsomeres 2–3; pulvillus well-developed, as long as well-developed claw, and as wide as base of claw; empodium absent.

##### Wing:

length = 10.2–13.3(–14.2) mm; hyaline throughout, slightly brown stained along veins, veins light brown, microtrichia absent; cells r_1_, r_4_, r_5_, m_3_, + cu*p* closed; C well-developed, around entire wing; R_4_ terminates in R_1_; R_5_ terminates in R_1_; stump vein (R_3_) at base of R_4_ present, long but not reaching R_2_; R_4_ and R_5_ widest apart medially; r-m distinct, R_4_+5 and M_1_ apart, connected by crossvein; M_1_ straight at r-m (not curving anteriorly), M_1_ (or M_1_+M_2_) terminates in C; CuA_1_ and CuA_2_ split proximally to m-cu (cell m_3_ narrow proximally); M_3_+CuA_1_ terminate together in C; A_1_ undulating, cell a_1_ wide, A_1_ and wing margin further apart proximally than distally; alula well-developed; halter light yellow.

##### Abdomen:

brown; setation comprised of scattered white setae, surface entirely smooth; T1 brown, narrow yellow posterior margin, T2–7 brown, broad yellow posterior margin, expanding antero-laterally particularly on T2–3; T1 long white setose, T2–3 sparsely white setose; T predominantly apubescent; S1 yellow, S2–7 yellow with brown areas medially and laterally; S1–3 asetose; S predominantly apubescent; T2–4 parallel-sided and not constricted waist-like; bullae on T2 black, transversely elongate, surface entirely smooth, T2 surface anterior to bullae smooth.

##### Male terminalia:

T1–7 well-developed, entirely sclerotised, T8 postero-medially weakly sclerotised, with anterior transverse sclerotised bridge connecting lateral sclerites; T7–8 anteriorly with 2 lateral apodemes; S6 regular, without any special setation postero-medially, S8 well-developed and simple, fused to T8 dorso-laterally, entire (undivided) ventro-medially; epandrium formed by single sclerite (fused medially ± entirely), pointed postero-laterally; subepandrial sclerite without lateral or median protuberances; hypandrium ± flat, rectangular to square sclerite, entirely fused with gonocoxite, forming a gonocoxite-hypandrial complex; gonocoxite dorso-ventrally flattened in distal ½, higher in proximal ½, with palp-like lateral appendage, gonocoxal apodeme present, short (at most slightly extending hypopygium anteriorly); 1 functional aedeagal prong, aedeagal epimere absent; lateral ejaculatory process absent; ejaculatory apodeme formed by single dorso-ventrally oriented plate; ventro-median margin of dorsal aedeagal sheath heavily sclerotised (appearing entirely closed); dorsal aedeagal sheath long, sperm sac entirely covered; sperm sac appearing ± heavily sclerotised.

**Figures 31–34.**
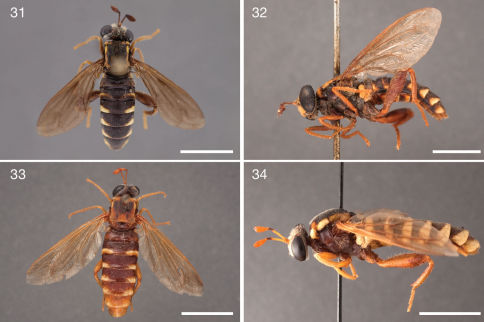
Photographs of Ectyphus pinguis specimens. **31** Ectyphus pinguis (♂, AAM-003496, AMGS, Morphbank) **32** Ectyphus pinguis (♂, AAM-003487, SAMC, Morphbank) **33** Ectyphus pinguis (♀, AAM-003509, SAMC, Morphbank) **34** Ectyphus pinguis (♂ holotype Ectyphus pinguis var. ceramiiformis, NMSA, Morphbank). Scale lines = 5 mm.

**Figures 35–38.**
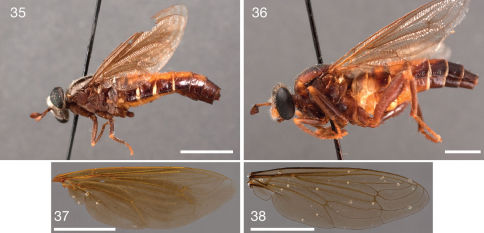
Photographs of Ectyphus pinguis specimens and wings of Ectyphus pinguis and Parectyphus namibiensis. **35** Ectyphus pinguis (♀ holotype Ectyphus bitaeniatus, NMSA, Morphbank) **36** Ectyphus pinguis (♀ holotype Ectyphus flavidorsalis, NMSA, Morphbank); Wings **37** Ectyphus pinguis (AAM-003463, NMSA, Morphbank) **38** Parectyphus namibiensis (AAM-003506, NMNW, Morphbank). Scale lines = 5 mm.

#### Re-description female:

##### Head:

brown, facial gibbosity yellow; mystax white, sparse short setae covering entire facial gibbosity; vertex predominantly apubescent, only lateral margin grey pubescent; maxillary palpus light brown.

##### Thorax:

brown; scutum surface entirely smooth, scutal setation comprised of distinct rows of long dorsocentral setae and lateral scutal setae.

##### Leg:

femur light brown.

##### Wing:

length = 12.0–13.7 mm.

##### Female genitalia:

densely arranged anteriorly directed setae absent, only few on T7–8 and S7–8; T8 with broad anterior rectangular apodeme; T9 formed by wide, rectangular sclerite with median protuberance; T9+10 entirely fused, T10 divided into 2 heavily sclerotised acanthophorite plates, 11 acanthophorite spurs per plate; 3 spermathecae, all equally large, formed by ± expanded weakly sclerotised ducts; individual spermathecal duct long; S9 (furca) formed by 2 sclerites, separated anteriorly and posteriorly, anterior furcal apodeme present, 2 lateral projections forming divided apodeme, lateral furcal apodeme absent, median furcal bridge absent.

#### Material examined:

**South Africa:** 1♂ ‚Africa australis‘, Drège (lectotype, ZMHB); 1F‚ Capland‘, Drège (paralectotype, ZMHB); Eastern Cape Province: 1♂ Cuylerville, 31°47'00"S; 26°56'00"E, 20.xii.1920, H. Cronwright (AAM-003522, SAMC); 1♀ Resolution, 33°10'00"S; 26°37'00"E, 23.xii.1927, A. Walton (holotype Ectyphus bitaeniatus, NMSA); 1♂ Willowmore, 33°17'00"S; 23°29'00"E, -.-.-, J. Brauns (holotype Ectyphus pinguis var. ceramiiformis, NMSA); 2♂ Willowmore, -.-.-, J. Brauns (holotype and paratype Ectyphus pinguis var. karooensis, NMSA); 1♀ Willowmore, -.-.-, J. Brauns (AAM-003492, USNM); 1♀ Willowmore, 20.xii.1909, J. Brauns (paratype Ectyphus flavidorsalis, NMSA); 1♀ Albany District, 33°24'00"S; 26°32'00"E, -.xii.1949, R. Phillips (AAM-003473, BMNH); 1♀ Georgida, 33°27'00"S; 23°17'00"E, 5.i.1927, J. Brauns (holotype Ectyphus flavidorsalis, NMSA); 1♀ 1♂ Dunbrody, 33°28'00"S; 25°33'00"E, -.-.1897, O‘Neil (AAM-003509–AAM-003510, SAMC); 1♂ Kleinemonde, 33°31'39"S; 27°03'00"E, -.-.-, J. Cooper (AAM-003487, SAMC); 2♂ Kleinemonde, -.i.1891, M. White (AAM-003520–AAM-003521, SAMC); 1♀ Port Alfred, 33°36'00"S; 26°54'00"E, 17.ii.1955, F. Junor (paratype Ectyphus pinguis var. litoralis, SAMC); 1♂ Port Alfred, 9.i.1971, J. Londt (AAM-003503, AMGS); 1♀ Port Alfred (AAM-003508, SAMC); 1♀ 2♂ Riet River mouth, 33°36'00"S; 26°54'00"E, 17.xii.1971, D. Greathead (AAM-003470–AAM-003472, BMNH); 1♀ 3♂ Rietrivier mouth, near Port Alfred, 17.xii.1974, F. Gess (AAM-003493–AAM-003496, AMGS); 5♂ Rietrivier mouth, near Port Alfred, 29.xii.1973, F. Gess (AAM-003497–AAM-003501, AMGS); 2♂ Algoa Bay, 33°41'34"S; 25°56'39"E, 15.xii.1892, J. Brauns (ZSMC); 2♀ 2♂ Swartkops, Algoa Bay, 33°51'49"S; 25°36'06"E, 25.xi.1921, J. Brauns (AAM-003447, AAM-003459–AAM-003461, NMSA); 1♂ Swartkops, Algoa Bay, 25.xi.1921, J. Brauns (AAM-003466, MZLU); 1♂ Swartkops, Algoa Bay, -.i.1919, B. Krüger (DEIC); 3♀ Swartkops, Port Elizabeth, 20.xi.1919, B. Krüger (AAM-003448, AAM-003462–AAM-03463, NMSA); 1♀ Swartkops, Port Elizabeth, -.ii.1919, B. Krüger (AAM-003465, NMSA); 1♂ Swartkops, Port Elizabeth, 25.xi.1919, B. Krüger (AAM-003464, NMSA); 1♀ 1♂ Swartkops, Port Elizabeth, 25.xi.1919, B. Krüger (AAM-003467–AAM-003468, ISNB); 4♀ 6♂ Gamtoos River Mouth, Papiesfontein, 33°57'47"S; 25°01'46"E, -.i.1960, SAM Museum Staff (paratype Ectyphus pinguis var. litoralis, SAMC); 1♂ Van Staden‘s River Mouth, 33°58'00"S; 25°13'00"E, -.i.1960, SAM Museum Staff (paratype Ectyphus pinguis var. litoralis, SAMC); 1♀ 1♂ Port Elizabeth, 33°58'00"S; 25°35'00"E, 1.ii.1950, A. Brown (AAM-003450–AAM-003451, NMSA); 1♀ Port Elizabeth, 1.i.1971, M. Strydom (AAM-003034, SANC); 2♀ 3♂ Port Elizabeth, Cape Recife area, 34°01'14"S; 25°40'60"E, 22–27.xii.1985, J. Londt (AAM-003442–AAM-003446, NMSA); 1♂ Jeffrey‘s Bay, Humansdorp, 34°02'00"S; 24°46'00"E, 23.xii.1922, J. Brauns (AAM-003469, BMNH); 1♀ The Willows, Port Elizabeth, 34°02'00"S; 25°36'00"E, 28.xii.1970, M. Strydom (AAM-003489, SANC); 1♂ Jeffrey‘s Bay, 34°03'00"S; 24°55'00"E, 19.xii.1922, (DEIC); 9♀ 3♂ Jeffrey‘s Bay, -.i.1960, SAM Museum Staff (holotype and paratype Ectyphus pinguis var. litoralis, SAMC); 1♀ 1♂ Jeffrey‘s Bay, -.i.1960, SAM Museum Staff (paratype Ectyphus pinguis var. litoralis, ISNB); 1♀ 1♂ Jeffrey‘s Bay, -.-.-, (AAM-003828–AAM-003829, SMNS); Western Cape Province: 1♀ Tulbagh, 33°17'00"S; 19°09'00"E, 10.xii.1924, J. Brauns (AAM-003449, NMSA); 1♂ ‚Cape of Good Hope‘, 33°48'03"S; 19°00'36"E, -.-.1835, J. Verreaux (MNHN).

#### Type locality, distribution, and biodiversity hotspots:

The original type locality is ‚Africa australis‘ (South Africa). Following recommendation 76A.1.4. of the *International Code of Zoological Nomenclature* (4th edition) a new type locality is selected from within the range of the species. We hereby designate the Riet River mouth (33°36'00"S; 026°54'00"E), near Port Alfred, Eastern Cape, South Africa as the new type locality. Cape Floristic Region and Maputaland-Pondoland-Albany biodiversity hotspots.

#### Remarks:

Specimens identified as belonging to the three varieties of Ectyphus pinguis were examined, including a large series of paratypes of Ectyphus pinguis var. litoralis. These specimens were determined to represent colour and vestiture variation in Ectyphus pinguis, rather than belonging to distinct subspecies. The three names, Ectyphus pinguis var. litoralis, Ectyphus pinguis var. karooensis, and Ectyphus pinguis var. ceramiiformis, were proposed by Hesse to delimit infrasubspecific entities. Because these names were never adopted as valid for a species or subspecies, as was already pointed out by Bowden (1980: 326), the names are unavailable following the *International Code of Zoological Nomenclature* (4th edition, Article 45.6.4. and 45.6.4.1.). The unique female holotype of Ectyphus bitaeniatus and the two female type specimens of Ectyphus flavidorsalis were also examined. These specimens represent Ectyphus pinguis in our view and are here synonymised with this species*.* Similar colour variation as exhibited by these two species was observed in female specimens of Ectyphus pinguis and therefore does not characterise distinct species. Hesse (1969: 372) mentioned the ♀ specimen from Tulbagh in the Western Cape as probably being mislabelled. We have studied the specimen and agree with his identification as Ectyphus pinguis, but cannot add any information whether the locality is correct or not. This locality is far removed from any other locality in the eastern Western Cape and in the western Eastern Cape provinces (Fig. 45).

### Ectyphus pretoriensis

(Bezzi, 1924)

Figs 9–10 23–25 45

Ectyphus pretoriensis  (Bezzi, 1924) Hesse 1969: 377; Bowden 1980: 326.
Ectyphus armipes  subsp. *pretoriensis*Bezzi 1924: 197.

#### Diagnosis:

The species is distinguished from congeners by the brown metepimeron, the white setation on the head and scutum, the narrow posterior yellow margin on abdominal tergites, and its apparent distribution in Pretoria.

#### Re-description male:

##### Head:

black, facial gibbosity light brown, in general lightly silver pubescent; width distinctly greater than thorax, interocular distance on vertex larger than at ventral eye margin, vertex between compound eyes slightly depressed, parafacial area very narrow, facial gibbosity nearly touching median eye margin; facial gibbosity distinct, well-developed and discernible in lateral view; mystax white, covering entire facial gibbosity; frons entirely grey pubescent, vertex medially apubescent, laterally silver pubescent, postgena lightly silver pubescent; setation: vertex white, frons white, ocp setae white, pocl setae white; ocellar triangle apubescent; proboscis brown, short, about ½ length of oral cavity; labellum small, as wide as prementum, about ¾ length of prementum, unsclerotised laterally; maxillary palpus cylindrical, light brown, longer than ½ length of proboscis.

##### Antenna

brown, scape and pedicel white setose dorsally and ventrally; postpedicel cylindrical in proximal ½, symmetrically bulbous in distal ½, ≥ 5.0 times as long as combined length of scape and pedicel; apical ‚seta-like‘ sensory element situated apically in cavity on postpedicel.

##### Thorax:

brown, lightly grey pubescent; scutum medially dark brown, laterally brown, surface entirely smooth, lightly grey pubescent, scutal setation comprised of distinct rows of long dorsocentral setae and lateral scutal setae; dc setae pre- and postsuturally white, acr setae absent, lateral scutal setae white, npl, spal, and pal setae absent; postpronotal lobe light brown, grey pubescent; proepisternum, lateral postpronotum, and postpronotal lobe long white setose; scutellum lightly grey pubescent, long white setose, apical scutellar setae present; mesopostnotum, anatergite, and katatergite silver pubescent, asetose; katatergite elevated and smoothly convex; anterior anepisternum asetose, supero-posterior anepisternum asetose; posterior anepimeron long white setose, katepimeron asetose; metepimeron evenly elevated, same colour as T1, lightly silver pubescent, asetose; metepisternum silver pubescent, asetose.

##### Leg:

light brown, setation predominantly white; pro, mes, and met coxa grey pubescent, white setose; met trochanter macrosetose medially; femur light brown, met femur evenly clubbed in distal ¾, in distal ½ macrosetose, 1 antero-ventral and 1 postero-ventral row of macrosetae; pro, mes, and met tibia straight, met tibia cylindrical with distinct ventral keel terminating into a sharp spine; pro and mes tarsomere 1 as long as combined length of tarsomeres 2–3, met tarsomere 1 as long as combined length of tarsomeres 2–3; pulvillus well-developed, as long as well-developed claw, and as wide as base of claw; empodium absent.

##### Wing:

length = 11.6 mm; hyaline throughout, veins light brown, microtrichia absent; cells r_1_, r_4_, r_5_, m_3_, + cu*p* closed; C well-developed, around entire wing; R_4_ terminates in R_1_; R_5_ terminates in R_1_; stump vein (R_3_) at base of R_4_ present, short not reaching R_2_; R_4_ and R_5_ widest apart medially; r-m distinct, R_4_+5 and M_1_ apart, connected by crossvein; M_1_ straight at r-m (not curving anteriorly), M_1_ (or M_1_+M_2_) terminates in C; CuA_1_ and CuA_2_ split proximally to m-cu (cell m_3_ narrow proximally); M_3_+CuA_1_ terminate together in C; A_1_ undulating, cell a_1_ wide, A_1_ and wing margin further apart proximally than distally; alula well-developed; halter light brown.

##### Abdomen:

brown; setation comprised of scattered white setae, surface entirely smooth; T1–7 brown with narrow light brown to dark yellow posterior margin; T1 long white setose, T2–3 sparsely white setose; T1–4 anteriorly lightly grey pubescent, T5–7 apubescent; S1 yellow, S2–7 brown with light brown posterior margin; S1 asetose, S2–3 sparsely white setose; S predominantly apubescent; T2–4 parallel-sided and not constricted waist-like; bullae on T2 brown, transversely elongate, surface entirely smooth, T2 surface anterior to bullae smooth.

##### Male terminalia:

T1–7 well-developed, entirely sclerotised, T8 postero-medially weakly sclerotised, with anterior transverse sclerotised bridge connecting lateral sclerites; T7–8 anteriorly with 2 lateral apodemes; S6 regular, without any special setation postero-medially, S8 well-developed and simple, fused to T8 dorso-laterally, entire (undivided) ventro-medially; epandrium formed by single sclerite (fused medially ± entirely), pointed postero-laterally; subepandrial sclerite without lateral or median protuberances; hypandrium ± flat, rectangular to square sclerite, entirely fused with gonocoxite, forming a gonocoxite-hypandrial complex; gonocoxite dorso-ventrally flattened in distal ½, higher in proximal ½, with palp-like lateral appendage, gonocoxal apodeme present, short (at most slightly extending hypopygium anteriorly); 1 functional aedeagal prong, aedeagal epimere absent; lateral ejaculatory process absent; ejaculatory apodeme formed by single dorso-ventrally oriented plate; ventro-median margin of dorsal aedeagal sheath heavily sclerotised (appearing entirely closed); dorsal aedeagal sheath long, sperm sac entirely covered; sperm sac appearing ± heavily sclerotised.

#### Re-description female:

##### Head:

brown, facial gibbosity yellow; vertex entirely grey pubescent; maxillary palpus brown.

##### Antenna

scape and pedicel white and yellow setose dorsally and ventrally.

##### Thorax:

light brown; scutum yellow, broad brown median presutural stripe and brown paramedial postsutural stripes, surface entirely smooth; scutal setation comprised of distinct rows of short dorsocentral setae and lateral scutal setae; postpronotal lobe yellow, partly white pubescent; proepisternum, lateral postpronotum, and postpronotal lobe short white setose; metepimeron silver pubescent, asetose.

##### Leg:

brown and yellow; femur brown.

##### Wing:

length = 11.9 mm; halter light yellow.

##### Abdomen:

brown and yellow; T1 brown, T2 brown with yellow anterior and posterior margin, T3–7 brown laterally and yellow medially; T1–3 sparsely white setose; S1 yellow, S2–7 brown with scattered yellow areas; bullae on T2 black, transversely elongate.

##### Female genitalia:

densely arranged anteriorly directed setae absent, only few on T7–8 and S7–8; T8 with broad anterior rectangular apodeme; T9 formed by wide, rectangular sclerite with median protuberance; T9+10 entirely fused, T10 divided into 2 heavily sclerotised acanthophorite plates, 11 acanthophorite spurs per plate; 3 spermathecae, all equally large, formed by ± expanded weakly sclerotised ducts; individual spermathecal duct long; S9 (furca) formed by 2 sclerites, separated anteriorly and posteriorly, anterior furcal apodeme present, 2 lateral projections forming divided apodeme, lateral furcal apodeme absent, median furcal bridge absent.

#### Material examined:

**South Africa:** Gauteng Province: 1♂ Willows, Pretoria, 25°44'60"S; 28°20'47"E, 23.ix.1917, H. Munro (lectotype, SAMC); 1♀ 1♂ Willows, Pretoria, 23.ix.1917, H. Munro (NMSA); 1♀ Fairy Glen (= Faerie Glen), Pretoria, 25°46'24"S; 28°18'03"E, 19.ix.1915, H. Munro (paralectotype, SAMC); 1♂ Fairy Glen (= Faerie Glen), Pretoria, 19.ix.1915, H. Munro (NMSA).

#### Type locality, distribution, and biodiversity hotspot:

Willows (suburb of Pretoria), 25°44'60"S; 28°20'47"E, Gauteng, South Africa. Does not occur in any currently recognised biodiversity hotspot.

#### Remarks:

In order to preserve taxonomic stability and make more universal the use of this specific name, the ♂ specimen from Willows, is here designated as the lectotype, making the ♀ specimen from Faerie Glen a paralectotype.

### Genus Parectyphus

Hesse, 1972

Parectyphus Hesse 1972: 165. Type species: Parectyphus namibiensis Hesse, 1972, by monotypy.

#### Diagnosis:

The genus can be distinguished from other Afrotropical Mydidae by the presence of a complete stump vein (R_3_) connecting R_2_ and R_4_, the distinctly clubbed metathoracic femur, the presence of a ventral keel on the metathoracic tibia terminating into a well-developed apical spine, M_3_+CuA_1_ terminate together into C on the posterior wing margin, the configuration of the male terminalia, and its apparent distribution in south-western Namibia and far north-western South Africa.

### Parectyphus namibiensis

Hesse, 1972

Figs 26–28, 30 38 44 45

Parectyphus namibiensis Hesse 1972: 165; Bowden 1980: 326.

#### Diagnosis:

See above for generic diagnosis.

#### Re-description male:

##### Head:

black, facial gibbosity light brown, rarely black, in general rarely predominantly apubescent, yellow pubescent on median eye margin; width distinctly greater than thorax, interocular distance on vertex larger than at ventral eye margin, vertex between compound eyes slightly depressed, parafacial area very narrow, facial gibbosity nearly touching median eye margin; facial gibbosity distinct, well-developed and discernible in lateral view; mystax white, covering entire facial gibbosity, rarely yellow, covering entire facial gibbosity; frons predominantly apubescent, vertex medially apubescent, laterally grey pubescent, rarely medially apubescent, laterally yellow pubescent, postgena lightly silver pubescent; setation: vertex white, rarely yellow, frons white, rarely yellow, ocp setae white, rarely yellow, pocl setae white, rarely yellow; ocellar triangle apubescent; proboscis brown, long, projecting beyond fronto-clypeal suture; labellum small, as wide as prementum, as long as prementum, unsclerotised laterally; maxillary palpus cylindrical, brown, about ½ length of proboscis.

##### Antenna

brown, scape and pedicel brown setose dorsally and ventrally; postpedicel cylindrical in proximal ½, symmetrically bulbous in distal ½, ≥ 4.0 times as long as combined length of scape and pedicel, rarely ≥ 6.0 times as long as combined length of scape and pedicel; apical ‚seta-like‘ sensory element situated apically in cavity on postpedicel.

##### Thorax:

brown, rarely dark brown to bluish-black, scutum predominantly grey pubescent, pleura predominantly apubescent, rarely predominantly yellow pubescent; scutum medially bluish-black, laterally brown, surface entirely smooth, lightly grey pubescent, rarely lightly yellow pubescent, paramedial stripes (merging postsuturally) and posterior lateral stripes densely yellow pubescent, scutal setation comprised of scattered short white, sometimes black, setae with distinct rows of long dorsocentral setae and lateral scutal setae; dc setae pre- and postsuturally white, rarely pre- and postsuturally black, acr setae present, lateral scutal setae white, rarely black, npl, spal, and pal setae absent; postpronotal lobe light brown, partly silver pubescent, rarely dark brown, partly yellow pubescent; proepisternum, lateral postpronotum, and postpronotal lobe long brown setose, rarely long black setose; scutellum lightly grey pubescent, long white setose, rarely lightly grey pubescent, long black setose, apical scutellar setae present; mesopostnotum, anatergite, and katatergite grey pubescent, rarely lightly yellow pubescent, mesopostnotum asetose, anatergite long black setose or long white setose, katatergite asetose; katatergite elevated and smoothly convex; anterior anepisternum asetose, supero-posterior anepisternum long white setose, rarely long black setose; posterior anepimeron long black setose or long white setose, katepimeron asetose; metepimeron evenly elevated, same colour as T1, apubescent, asetose, rarely silver pubescent, asetose; metepisternum silver pubescent, asetose.

##### Leg:

brown, setation black and white; pro coxa apubescent, long black setose, mes coxa lightly silver pubescent, long white and black setose, rarely apubescent, long black setose, met coxa lightly silver pubescent, long white and black setose, rarely apubescent, long black setose; met trochanter setose medially or macrosetose medially; femur brown, met femur evenly clubbed in distal ¾, in distal ½ macrosetose, 1 antero-ventral and 1 postero-ventral row of macrosetae; pro, mes, and met tibia straight, met tibia cylindrical with distinct ventral keel terminating into a sharp spine; pro and mes tarsomere 1 about as long as individual tarsomeres 2, 3, or 4, met tarsomere 1 slightly longer than tarsomere 2, tarsomeres 1 and 2 longer than tarsomeres 3 and 4 combined; pulvillus well-developed, as long as well-developed claw, and as wide as base of claw; empodium absent.

##### Wing:

length = 13.5–15.5 mm; generally hyaline, sometimes slightly brown stained along veins, veins light brown, microtrichia absent; cells r_1_, r_4_, r_5_, m_3_, + cu*p* closed; C well-developed, around entire wing; R_4_ terminates in R_1_; R_5_ terminates in R_1_; stump vein (R_3_) at base of R_4_ present, long and connecting R_4_ and R_2_; R_4_ and R_5_ widest apart medially; r-m distinct, R_4_+5 and M_1_ apart, connected by crossvein; M_1_ straight at r-m (not curving anteriorly), M_1_ (or M_1_+M_2_) terminates in C; CuA_1_ and CuA_2_ split proximally to m-cu (cell m_3_ narrow proximally); M_3_+CuA_1_ terminate together in C; A_1_ undulating, cell a_1_ wide, A_1_ and wing margin further apart proximally than distally; alula well-developed; halter light brown, rarely brown.

##### Abdomen:

brown to bluish-black; setation comprised of scattered white and black setae, surface microrugose; T1–7 brown, rarely dark brown with narrow light brown posterior margin; T1 long white setose, T2–3 short black setose, rarely T1 long black setose, T2–3 short black setose; T predominantly apubescent; S1 light brown, S2–7 light brown with narrow yellow posterior margin, rarely S1–7 brown; S1 asetose, S2–3 short black setose; S predominantly apubescent; T2–4 parallel-sided and not constricted waist-like; bullae on T2 black, transversely elongate, surface entirely smooth, T2 surface anterior to bullae smooth ;.

##### Male terminalia:

T1–7 well-developed, entirely sclerotised, T8 postero-medially weakly sclerotised, with anterior transverse sclerotised bridge connecting lateral sclerites; T7–8 anteriorly with 2 lateral apodemes; S6 regular, without any special setation postero-medially, S8 well-developed and simple, fused to T8 dorso-laterally, entire (undivided) ventro-medially; epandrium formed by single sclerite (fused medially ± entirely), pointed postero-laterally; subepandrial sclerite without lateral or median protuberances; hypandrium ± flat, rectangular to square sclerite, entirely fused with gonocoxite, forming a gonocoxite-hypandrial complex; gonocoxite dorso-ventrally flattened in distal ½, higher in proximal ½, with palp-like lateral appendage, gonocoxal apodeme present, short (at most slightly extending hypopygium anteriorly); 1 functional aedeagal prong, aedeagal epimere absent; lateral ejaculatory process absent; ejaculatory apodeme formed by single dorso-ventrally oriented plate; ventro-median margin of dorsal aedeagal sheath heavily sclerotised (appearing entirely closed); dorsal aedeagal sheath long, sperm sac entirely covered; sperm sac appearing ± heavily sclerotised.

**Figures 39–44.**
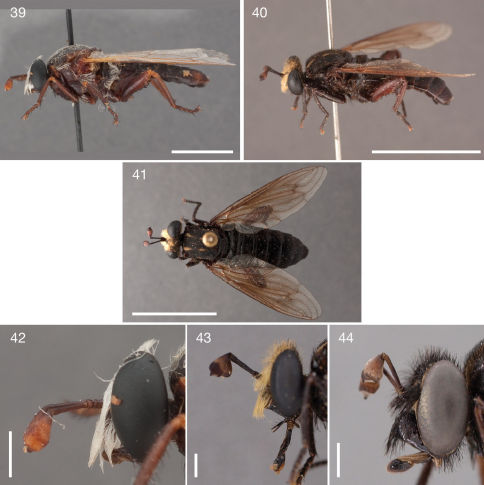
Photographs of Parectyphus namibiensis specimens. **39 + 42** Parectyphus namibiensis (♂ holotype, SMNS) **39** lateral (Morphbank) **42** head lateral (Morphbank); **40, 41, + 43** Parectyphus namibiensis (♂, AAM-003485, SAMC) **40** lateral (Morphbank) **41** dorsal (Morphbank) **43** head lateral (Morphbank) **44** Parectyphus namibiensis (♂, AAM-003606, NMNW, Morphbank). Scale lines = 5 mm.

#### Description female:

##### Head:

in general yellow pubescent; mystax black, covering entire facial gibbosity; frons yellow pubescent, vertex medially apubescent, laterally yellow pubescent; setation: vertex black, frons black, ocp setae black, pocl setae black.

##### Antenna

postpedicel ≥ 5.0 times as long as combined length of scape and pedicel.

##### Thorax:

light brown, predominantly yellow pubescent; scutum medially brown, laterally dark yellow, surface entirely smooth, lightly yellow pubescent, anterior paramedial and lateral stripes densely yellow pubescent; dc setae pre- and postsuturally black, acr setae present, rarely absent; lateral scutal setae black; postpronotal lobe light brown, partly white pubescent; proepisternum, lateral postpronotum, and postpronotal lobe long black setose; scutellum lightly grey pubescent, long black setose; mesopostnotum, anatergite, and katatergite lightly yellow pubescent, mesopostnotum asetose, anatergite long black setose; supero-posterior anepisternum long black setose; posterior anepimeron long black setose; metepimeron same colour as T1, lightly silver pubescent, asetose; metepisternum silver pubescent, asetose.

##### Leg:

brown and yellow, setation predominantly black; pro, mes, and met coxa apubescent, long black setose; met trochanter macrosetose medially; femur brown, met femur yellow; pro and mes tarsomere 1 longer than tarsomere 2, but less than combined length of tarsomeres 2–3; met tarsomere 1 as long as combined length of tarsomeres 2–3.

##### Wing:

length = 12.8–14.2 mm; slightly brown stained throughout; halter light brown.

##### Abdomen:

brown; setation comprised of dense short black setae; T1 brown, yellow medially, T2–7 brown with narrow dark yellow anterior and posterior margin, rarely T1–7 yellow, brown postero-laterally; T1–3 densely black setose; S1 light brown, S2–7 light brown with narrow yellow posterior margin.

##### Female genitalia:

densely arranged anteriorly directed setae present on T7–8 and S7–8; T8 with broad anterior rectangular apodeme; T9 formed by wide, rectangular sclerite with median protuberance; T9+10 entirely fused, T10 divided into 2 heavily sclerotised acanthophorite plates, 8 acanthophorite spurs per plate; 3 spermathecae, all equally large, formed by ± expanded weakly sclerotised ducts; individual spermathecal duct long; S9 (furca) formed by 2 sclerites, separated anteriorly and posteriorly, anterior furcal apodeme present, 2 lateral projections forming divided apodeme, lateral furcal apodeme absent, median furcal bridge absent.

#### Material examined:

**Namibia:** Erongo Region: 1♂ Gobabeb, 23°33'37"S; 15°02'26"E, 408 m, -.-.- (holotype, SMNS); Karas Region: 1♀ Kanaän 104, 25°50'42"S; 16°09'30"E, 6–7.x.1972, (AAM-003505, NMNW); 1♂ Klinghardt Mountains, 27°20'00"S; 15°45'00"E, 1.x.1982, V. Whitehead (AAM-003486, SAMC); 1♂ Klinghardt Mountains, 24.x.1977, V. Whitehead (AAM-003485, SAMC); 1♂ Namuskluft 88, 27°48'00"S; 16°52'00"E, 7–14.x.1970 (AAM-003504, SAMC); 3♀ 1♂ Rosh Pinah, 10 km NW, 27°54'00"S; 16°42'00"E, 13.viii.1990, C. Roberts E. Marais (AAM-003606–AAM-003609, NMNW); **South Africa:** Northern Cape Province: 2♀ Tnong-Gys Dunes, 29°32'50"S; 17°14'03"E, 23–25.ix.1988, J. Irish E. Marais (AAM-003506–AAM-003507, NMNW).

#### Type locality, distribution, and biodiversity hotspot:

Gobabeb (23°33'37"S; 15°02'26"E), Erongo, Namibia. Namibia and South Africa. Succulent Karoo biodiversity hotspot.

#### Remarks:

**Figure 45.**
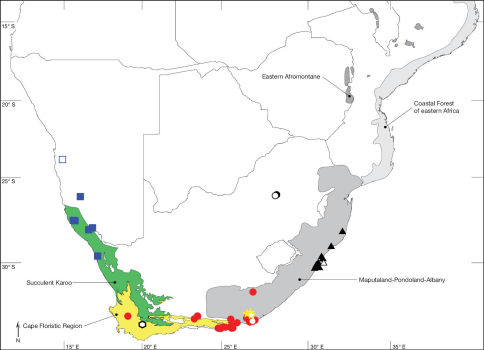
Map of southern Africa with biodiversity hotspots *sensu* Conservation International showing distribution of Ectyphus abdominalis (hexagon), Ectyphus armipes (triangle), Ectyphus capillatus (yellow star), Ectyphus pinguis (red circle), Ectyphus pretoriensis (black circle), and Parectyphus namibiensis (blue square). Type localities with open symbols.

Prior to this study, Parectyphus namibiensis was only known from the ♂ holotype collected at Gobabeb (Fig. 45). The location of new Parectyphus specimens in two museum collections has allowed us to describe the female for the first time and also to observe a considerable amount of colour variation among the 11 studied specimens. We dissected the ♂ terminalia from all localities, if available, to verify whether there is morphological variation in these generally species-specific features. However, we were unable to detect any differences and therefore concluded that all specimens represent Parectyphus namibiensis albeit coming from isolated collecting events (Fig. 45). The type locality, which marks the northern-most record, is some 700 km separated from the Tnong-Gys dunes in north-western South Africa as the southern-most record. While the holotype is entirely white setose (Fig. 39, 42), the specimens from the Klinghardt Mountains and Namuskluft, in the centre of the currently known distribution, are yellow setose (Fig. 40–41, 43) and the specimens from Rosh Pinah and Tnong-Gys dunes in the south are black setose (Fig. 44). The pubescence pattern on the scutum is in several instances very difficult to observe and since most specimens we studied were caught in wet Malaise traps, the coloration of the entire body might also differ among populations. Although Hesse (1972) observed an absence of macrosetae on the metathoracic trochanters of the holotype, the other specimens studied by us exhibit macrosetae, which is consistent with the delimitation of Ectyphinae as having macrosetose metathoracic trochanters (e.g., Wilcox and Papavero 1971), which separates this taxon from other Mydidae taxa. As mentioned under Ectyphus armipes, there are three specimens of this species known from Tongaat on the KwaZulu-Natal coast of South Africa in which the particular wing venation of Parectyphus, namely the stump vein (R_3_) entirely connecting R_2_ and R_4_ (Fig. 38), is also found. Parectyphus namibiensis exhibits other characteristics not found among Ectyphus species, e.g., the configuration of the male terminalia and the shape of the furca and spermathecae in the females, and therefore we do not propose to synonymise both genera. A comprehensive phylogenetic analysis of Mydidae, including all Ectyphinae, genera currently in preparation by the second author, will shed light on this question and establish whether Ectyphus and Parectyphus are adelphotaxa or whether Parectyphus is just an apomorphic Ectyphus.

## Identification key to the genera and species of Ectyphinae

A dichotomous identification key to all species of Ectyphus and Parectyphus including the three remaining species from the Nearctic Region is provided below. An illustrated online version of this key can be accessed at (http://www.mydidae.tdvia.de/online_keys) and a multi-access, matrix-based key can be accessed on the same web-site. An updated, illustrated identification key to all 11 currently recognised subfamily taxa, which is based on the key by Papavero and Wilcox (1974), can also be accessed on the above web-site.

**Table:** 

1	Metathoracic femur cylindrical, only slightly wider than prothoracic and mesothoracic femur; postpedicel bulbous in distal ½ (cylindrical in proximal ½); Nearctic	8
–	Metathoracic femur distinctly clubbed, much wider than prothoracic and mesothoracic femur (Fig. 5); postpedicel bulbous in distal ⅔ (cylindrical in proximal ⅓); Afrotropical (Fig. 1)	2
2	Stump vein (R_3_) connecting R_4_ and R_2_ (Fig. 38); anatergite setose (Fig. 39); supero-posterior anepisternum setose (Fig. 39); discal scutellar and apical scutellar setae present; proboscis long, rojecting well beyond fronto-clypeal suture (Fig. 40); ♂ aedeagus large, laterally compressed; ♀ ovipositor with 8 acanthophorite spurs per plate	Parectyphus namibiensis Hesse, 1972
–	Stump vein (R_3_) extending from R_4_, but not reaching R_2_ (Fig. 37); anatergite asetose (Fig. 33); supero-posterior anepisternum asetose (Fig. 36); discal scutellar and apical scutellar setae usually absent, rarely laterally developed; proboscis short (Fig. 32), only in a single species projecting slightly beyond fronto-clypeal suture (Fig. 6); ♂ aedeagus rounded, with a cup-like opening; ♀ ovipositor with 11 acanthophorite spurs per plate	3
3	Facial gibbosity yellow (Fig. 32); postpronotal lobes yellow (Fig. 34)	5
–	Facial gibbosity brown; postpronotal lobes brown (Fig. 5)	4
4	Proboscis about ½ as long as oral cavity; labellum much shorter than prementum; parafacial area narrow, facial gibbosity nearly touching median eye margin; vertex and frons light brown setose; western South Africa (Montagu)	Ectyphus abdominalis Bezzi, 1924
–	Proboscis slightly longer than oral cavity (Fig. 6); labellum as long as prementum; parafacial area wide, facial gibbosity in anterior view clearly separated from median eye margin; vertex and frons white setose; eastern South Africa (along KwaZulu-Natal coast)	Ectyphus armipes Bezzi, 1924
5	T5–7 densely long white setose (Fig. 8); abdominal tergites microrugose (setae with distinct sockets, especially where brown coloured)	Ectyphus capillatus Hesse, 1969
–	T5–7 sparsely short white setose (Fig. 4); abdominal tergites entirely smooth	6
6	Katatergite yellow; legs yellow to light brown (Fig. 4); T1–7 with entire yellow posterior margins (Figs 3–4); eastern Africa (Kenya)	Ectyphus amboseli sp. n.
–	Katatergite brown (Fig. 9); legs primarily brown (Fig. 9); T1–7 at least medially with brown posterior margin (some specimens with narrow yellow posterior margins, Fig. 31); South Africa	7
7	Metepimeron yellow (in stark contrast to brown T1, Fig. 32); ♂ palp-like lateral gonocoxal appendage situated sub-apically (Fig. 20); ♂ and ♀ abdominal tergites with broad yellow latero-posterior margins (sometimes yellow margins occupy latero-posterior ½ of tergite, Figs 31, 33); south to south-western South Africa (Eastern and Western Cape, Fig. 45)	Ectyphus pinguis Gerstaeckerр 1868
–	Metepimeron brown (same colour as T1, Fig. 9); ♂ palp-like lateral gonocoxal appendage situated apically (Fig. 23); ♂ and ♀ abdominal tergites with very narrow yellow latero-posterior margins (Figs 9–10); north-eastern South Africa (Gauteng, Fig. 45)	Ectyphus pretoriensis (Bezzi, 1924)
8	Anepisternum and katepisternum partly grey pubescent (not obvious to naked eye); R_5_ terminates in C (cell R_4_ open), sometimes in R_1_ very close to C; apical metathoracic tibial spine present in ♂ and ♀; ♂ hypandrium partially fused to gonocoxite; ♂ palp-like lateral gonocoxal appendage absent	Heteromydas bicolor Hardy, 1944
–	Dorsal anepisternum and dorsal katepisternum distinctly white pubescent (visible to naked eye); R_5_ terminates in R_1_ (cell R_4_ closed); apical metathoracic tibial spine present in ♂, absent or very much reduced in ♀; ♂ hypandrium completely free from gonocoxite; ♂ palp-like lateral gonocoxal appendage present	9
9	Supero-posterior anepisternum with only few white setae; bulbous (distal) part of postpedicel sub-equal to or longer than cylindrical (proximal) part; thorax generally yellow to light brown; ♂ abdominal tergites lightly grey pubescent; for ♂ terminalia see Kondratieff and Fitzgerald 1996	Opomydas limbatus Williston, 1886
–	Supero-posterior anepisternum densely long white setose; bulbous (distal) part of postpedicel shorter than cylindrical (proximal) part; thorax generally dark brown; ♂ abdominal tergites apubescent; for ♂ terminalia see Kondratieff and Fitzgerald 1996	Opomydas townsendi Williston, 1898

## Discussion

### Biology and ecology

The family Mydidae is most often collected in arid to semi-arid areas, and Ectyphus and Parectyphus are not exceptional. Hesse described the then known habitats as “scrub- and sclerophyll-covered dunes” (1969: 372) and “semi-wooded and forested parts” (1972: 165) of southern Africa. Even in the Namib Desert, the holotype of Parectyphus namibiensis was probably collected from a “wooded environment ... namely that found along the banks of the Kuiseb River” (Hesse 1972: 165). The new collecting localities for Parectyphus namibiensis (Fig. 45) occur inland, but sand dunes or at least dry, sandy river beds are present. Several species of Ectyphus have been collected at river mouths with presumably larger amounts of open sand, *i.e.*, Gamtoos River near Papiesfontein, Riet River in Port Alfred, Van Staden‘s River, and possibly the Tongaat River. According to Hesse (1969) Ectyphus is often collected resting on the ground or sand in open spaces. Although the life history of species of Ectyphinae has not been observed, behavioural characteristics are probably similar to other Mydidae. All species appear to have functional mouthparts, although these can be short as in Ectyphus abdominalis, Ectyphus amboseli, Ectyphus capillatus, Ectyphus pinguis, and Ectyphus pretoriensis, and so probably visit flowers and feed on pollen and nectar. The larvae of Ectyphus and Parectyphus remain unknown.

### Seasonal incidence

Ectyphus: Ectyphus abdominalis: January; Ectyphus amboseli sp. n.: September; Ectyphus armipes: November–April, July, September; Ectyphus capillatus: December–January; Ectyphus pinguis: November–February; Ectyphus pretoriensis: September; Parectyphus: Parectyphus namibiensis: August–October. While Ectyphus armipes appears to fly for much of the year along the KwaZulu-Natal coast, Ectyphus abdominalis, Ectyphus capillatus, and Ectyphus pinguis appear only during the southern Hemisphere summer and both, Ectyphus pretoriensis and Parectyphus namibiensis, fly only in spring.

### Biodiversity hotspots

Areas of high plant endemism, which are under serious threat of destruction and which have already sustained loss of biodiversity, are referred to as biodiversity hotspots by Conservation International (http://www.conservation.org) (Myers et al. 2000). The presence or absence of Mydidae species in designated biodiversity hotspots is an indication of whether these species will be protected when funding for the preservation of the hotspots is made available. Ectyphus armipes and Ectyphus capillatus are endemic to the Maputaland-Pondoland-Albany hotspot. Ectyphus pinguis is found in both Maputaland-Pondoland-Albany and the Cape Floristic Region, as well as slightly outside of both of these hotspots. Ectyphus abdominalis is endemic to the Cape Floristic Region. Parts of the range of Parectyphus namibiensis are in the Succulent Karoo hotspot. Ectyphus amboseli sp. n. was collected just outside the boundaries of the Eastern Afromontane hotspot, but further collection efforts in localities with higher elevations may result in Ectyphus amboseli specimens from this patchy biodiversity hotspot. The majority of species and specimens studied occur or are endemic to biodiversity hotspots *sensu* Conservation International. However, two species, Ectyphus amboseli sp. n. and Ectyphus pretoriensis, do not occur in any biodiversity hotspot.

## Conclusion

The description of Ectyphus amboseli sp. n. expands the distribution of Afrotropical Ectyphinae by presenting a Kenyan species far from all other known species in southern Africa. This distribution does also have implications for the discussion of the phylogenetic relationships of Ectyphinae to other Mydidae, which are being further investigated by the second author (in prep.). In addition to the previously known distribution in southern Africa and western North America, Ectyphinae are now also known from eastern Africa. A similar distribution with a western North American element and a primarily southern African element within the Afrotropical Region is also found within the Willistonininae of the Asilidae (Dikow 2009). Within Willistonininae, the genus Sisyrnodytes Loew, 1856 even occurs in the southern Palaearctic Region, but the highest species diversity is found in southern Africa (see Londt 2009). We predict that additional records of Ectyphus, or possibly even undescribed species, will become available with more field work along the eastern African coast and especially in Mozambique and Tanzania, both poorly collected areas.

## Supplementary Material

XML Treatment for Ectyphus

XML Treatment for Ectyphus abdominalis

XML Treatment for Ectyphus amboseli

XML Treatment for Ectyphus armipes

XML Treatment for Ectyphus capillatus

XML Treatment for Ectyphus pinguis

XML Treatment for Ectyphus pretoriensis

XML Treatment for Parectyphus

XML Treatment for Parectyphus namibiensis
